# Canopeo app as image-based phenotyping tool in controlled environment utilizing Arabidopsis mutants

**DOI:** 10.1371/journal.pone.0300667

**Published:** 2024-03-21

**Authors:** Gabriella Hale, Ning Yuan, Lavanya Mendu, Glen Ritchie, Venugopal Mendu

**Affiliations:** 1 Department of Plant and Soil Science, Fiber and Biopolymer Research Institute (FBRI), Texas Tech University, Lubbock, Texas, United States of America; 2 Department of Plant Science and Plant Pathology, Montana State University, Bozeman, Montana, United States of America; 3 Department of Plant and Soil Science, Texas Tech University, Lubbock, Texas, United States of America; Central University of Haryana School of Life Sciences, INDIA

## Abstract

Canopeo app was developed as a simple, accurate, rapid, and free tool to analyze ground cover fraction (GCF) from red-green-blue (RGB) images and videos captured in the field. With increasing interest in tools for plant phenotyping in controlled environments, the usefulness of Canopeo to identify differences in growth among *Arabidopsis thaliana* mutants in a controlled environment were explored. A simple imaging system was used to compare Arabidopsis mutants based on the FLAVIN-BINDING, KELCH REPEAT, F-BOX-1 (FKF1) mutation, which has been identified with increased biomass accumulation. Two FKF1 lines such as null expression (*fkf1-t*) and overexpression (FKF1-OE) lines were used along with wild type (Col-0). Canopeo was used to phenotype plants, based on biomass estimations. Under long-day photoperiod, *fkf1-t* had increased cellulose biosynthesis, and therefore biomass. Resource partitioning favored seedling vigor and delayed onset of senescence. In contrast, FKF1-OE illustrated a determinative growth habit where plant resources are primarily allocated for seed production. This study demonstrates the use of Canopeo for model plants and highlights its potential for phenotyping broadleaved crops in controlled environments. The value of adapting Canopeo for lab use is those with limited experience and resources have access to phenotyping methodology that is simple, accessible, accurate, and cost-efficient in a controlled environment setting.

## Introduction

Collecting phenotypic data, such as biomass accumulation rate, in controlled environments requires sampling many replicates in a reproducible manner. In field studies, plant communities demonstrate variability within the population while in a controlled environment this variability is reduced because plants were studied as single specimens for observing individual changes. With the rise of ground cover fraction (GCF) analysis from visible spectrum image sensors, remote sensing has gained a technological edge as a phenotyping tool with the potential to save time and money [1]. Remote sensing plant populations is advantageous because data can be collected non-invasively over large areas with less time and less labor inputs than in traditional methods [2–4]. Visible spectrum image sensors collect light in the visible spectrum wavelengths, typically in the wavelength range of 400 to 780 nm. Images are created from visible reflected light in red, green, and blue (RGB) wavelengths and serve to digitally visualize morphometric parameters [5], plant growth, and biomass [6] after image acquisition and processing. Advantages of RGB sensors include low-cost, less time, simple, and automation of data acquisition relative to manual field monitoring.

In addition to the use of individual spectral bands, multi-spectral and hyperspectral image sensors can provide more detailed spectral information. Multi-spectral image sensors provide canopy reflectance data using three to ten spectral bands that can be calculated into “Normalized Difference Vegetation Index” (NDVI) data, which has demonstrated a strong correlation (r^2^ = 0.87 to 0.95) with ground measurements [7,8]. Hyperspectral images can provide highly accurate spectral information for plant stress analysis [9,10] and overall plant assessment [11] because hundreds of narrow spectral bands are used during image acquisition. Hyperspectral image sensors are more accurate than multispectral or single-band image sensors. However, obstacles such as price of the sensors, image processing, and image analysis have limited the accessibility of both multispectral and hyperspectral sensors as a phenotyping tool [7,12]. Other sensor types for estimating plant biomass include depth image sensors and Light Detection and Ranging (LiDAR), which can used for 3-D reconstruction of plants to estimate plant traits [13,14].

Close-range remote sensing, the application of remote sensing tools at small spatial scales [15], includes imaging technologies that are not conventionally classified as remote sensing like close-range photogrammetry and terrestrial LiDAR. Close-range remote sensing can be used to enhance morphological and physiological measurement efficiency for individual plants, and it is a valuable tool for verifying sensing reliability at larger scales [16]. Plant traits can be measured in detail when plant to plant interactions are inferred at the individual level; while grouped plant individuals grown in controlled environment settings can be monitored with remote sensing tools at close range to measure relative changes in plant traits. By controlling the limiting factors of the artificial environment, an increase or decrease in expression of a particular trait can be observed among individual plants and plant-to-plant interactions. For example, hyperspectral close-range sensing has been used to collect detailed temporal data of defense responses of plants. This approach has overcome the drawback of invasively measuring induced defense response, thereby improving the reliability of collected data. Further, close-range remote sensing tools can be supplemented with other technologies to improve inference of phenotypic traits and plant-to-plant interactions.

The Canopeo app is a close-range remote sensing tool designed for processing RGB digital images of field crops captured with a smartphone into GCF measurements [17]. Canopeo measures estimated green canopy cover as a percentage of green pixels detected in the camera’s field of view. This measurement can be used directly for monitoring relative growth rates [18]. Through a simplified user interface, images captured with the smartphone are processed in the app to measure GCF which can then be directly used for expressing estimated green biomass accumulation as grams. Canopeo has been used as a high-throughput phenotyping tool for measuring biomass traits of sorghum cultivars [19] and shoot biomass of twenty lentil lines [20]. Canopeo’s other novel applications include its usefulness as a tool for sensor-based yield prediction [21–24], investigating biomass partitioning [25], developing critical nitrogen dilution curves [26], making nitrogen rate recommendations [21,26], determining appropriate time to start grazing in tropical pasture management [27], and improving weed management programs [28]. However, Canopeo was not found useful for visually assessing symptoms of Verticillium wilt in potato [29]. The accuracy of disease detection reportedly decreased as Verticillium wilt severity increased. Authors hypothesized that this result could be due to uneven progress of wilt spreading over time, since disease progresses slowly in the beginning and then rapidly peaks toward final stages of crop growth. Lastly, Canopeo is the only reported precision agriculture smartphone tool with the capacity to analyze GCF from videos [17,30]. A review article [30] on over thirty smartphone applications targeting precision agriculture practices highlights how the development of smartphone based agricultural applications has increased exponentially over time. For example, Onwuchekwa-Henry, C. B., et al. [22] combined predictors of Canopeo-derived canopy cover and weather to create a tool for identifying and managing fertilizer and water supply to maximize productivity in rice production systems. Thus, this paper is a resource to help researchers and farmers understand the limitations of the existing smartphone-based sensing tool, Canopeo, and where new contributions can be performed with its application in plant phenotyping.

These reports highlight Canopeo’s value as a novel tool for generating data beyond GCF. Imagination is the limiting factor for Canopeo’s diversity of applications. Thus, Canopeo measurements of estimated green biomass accumulation are hypothesized to be useful for creating a tool in a controlled lab environment for 1) identifying the variation of biomass accumulation and partitioning among natural or mutant populations, which could then aid in phenotyping and characterization of genotypes with genetic variations; and 2) determining whether biomass production is dependent upon (i.e., is a function of) stomatal density in natural or mutant populations. Reasoning for this is stomatal density is a component of stomatal conductance. Stomatal conductance, in turn, influences rate of CO_2_ fixation and thus biomass accumulation. Application of Canopeo for investigating relationships between reproductive and vegetative biomass partitioning and stomatal traits has not been previously reported.

In addition to estimating green biomass accumulation, Canopeo may be capable of detecting leaf necrosis rate in a controlled environment. Canopeo detects green light reflected off the plant, but once the plant enters senescence the green pigment in leaves begins to change from yellow to brown color. The more advanced senescence is, the more necrotic tissue will be present, lacking green pigment and decreasing biomass estimates of Canopeo [31]. This causes Canopeo to detect fewer green pixels in the digital image, thus resulting in a lower estimation of biomass accumulation. This does not indicate a decrease in biomass, rather, it visually indicates the rate at which senescence occurs for each genotype.

Here, the usefulness of Canopeo as a laboratory phenotyping tool is investigated by developing a simplistic methodology for imaging plants with a smartphone and five protocols for subsequent data analysis. The experimental approach served to determine Canopeo’s value for phenotyping biomass related traits of three *Arabidopsis thaliana* genotypes: wild-type (Col-0), mutant (*fkf1-t)*, and overexpression (FKF1-OE) lines all of which express different amounts of the blue-light photoreceptor protein, FLAVIN-BINDING KELCH REPEAT, F-BOX 1 (FKF1). To better understand the molecular mechanisms underpinning cellulose synthesis regulation in plants with FKF1 mutation, measurements were captured at the cellular level and whole-plant level using Canopeo and stomatal density samples.

The overall goal of this research was to develop a simple data collection methodology to test Canopeo’s phenotyping performance in a controlled lab environment and compare it to Canopeo’s phenotyping performance in field and greenhouse environments [17,19,20,24,32–35]. With this in mind, the objectives were to 1) develop a cost-efficient imaging system and experimental protocol for rapidly adapting Canopeo to lab use; 2) test Canopeo-MATLAB for its usefulness in phenotyping seedling biomass accumulation traits via batch processing of images; and 3) test the Canopeo phone app for its role in phenotyping the following traits across developmental stages: above-ground biomass accumulation, early vigor, onset of senescence, stomatal density and leaf area, with the secondary purpose of guiding hypotheses for additional roles of the FKF1 protein.

## Materials and methods

To test if Canopeo is useful in a controlled environment for phenotyping experiments, Canopeo measurements of estimated biomass accumulation of six developmental stages were associated with dry biomass weights, early vigor, onset of senescence, stomatal density, and leaf area using three Arabidopsis genotypes (mutant, overexpression line and Col-0). An imaging system and experimental protocol was designed for adapting Canopeo in lab environment. Experiments calibrating Canopeo GCF measurements with dry biomass weights served to validate the relationship between Canopeo and biomass accumulation estimation as grams. Experiments analyzing Canopeo data using SAS® Glimmix (Version 8.3) [36] has served to determine genotypic interactions with estimate biomass accumulation measured at different developmental stages. Experiments correlating estimate biomass accumulation and stomatal density has served to determine the relationship between biomass accumulation, stomatal density, and stomatal conductance. Estimating stomatal frequency served to validate the findings of this experiment.

### Plant material

The FKF1 mutant (*fkf1-t*) and Col-0 plants were obtained from TAIR and characterized as described [37]. Col-0 was used as an experimental control. The FKF1 overexpression line (FKF1-OE) plants were obtained from the Mendu Lab and characterized as described [38]. Plants were grown in controlled environment plant growth chambers (Percival growth chamber; Percival Intellus control system), at a temperature of 22°C. All plants were grown under long photoperiod conditions (16 hours day and 8 hours night cycle). Seedlings were transplanted in individual pots between 7 and 10 days after germination and similar sized plants were used for the experiments. Plants were fertilized with granular slow-release fertilizer (14-14-14 NPK) immediately after transplanting at a rate of 4 mL per growth flat. Randomized complete block design was used for growing these plants in flats. Each flat had 18 pots with one plant per pot. A total of 36 (+/- 5) plants were grown in two flats and used for one independent experiment, with approximately 12 plants per genotype. Low light intensity environment was provided in the growth chamber lighting using a spectrum of red + blue + white LEDs with a photosynthetic photon flux density (PPFD) of 36 μmol/m^2^/s. While high light intensity environment was provided in the growth chamber lighting using a spectrum of red + blue + white LEDs with a PPFD of 298 μmol/m^2^/s.

### Canopeo imaging and testing

**Fig 1:**
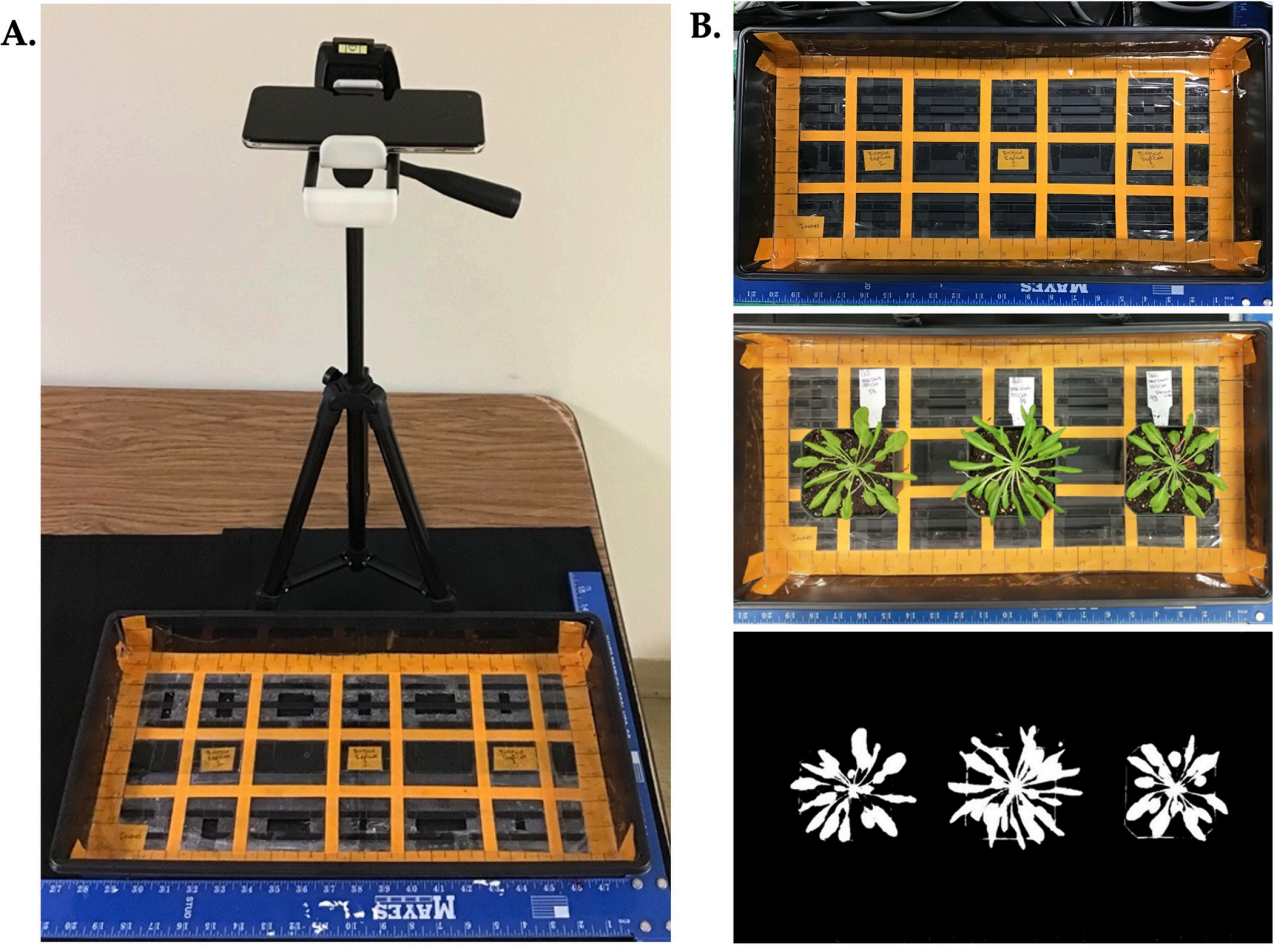
Image station setup for Canopeo imaging under lab environment. (A) A tripod is set up at 64 cm (25 inches) height to take images using cellphone with Canopeo app. Tripod feet were centered behind the tray, using the square ruler as a guide for repeatable placement. (B) A dark colored background was used as an imaging backdrop. A dark colored tray with gridlines was used for the repeatable spacing of specimens. Digital images were uploaded to the Canopeo app using the smartphone.

A standard imaging system was developed that allowed images to be taken with the same field of view and color characteristics. A dark colored imaging backdrop (Fig 1A) was used to minimize background noise of green reflected light. A dark colored tray with marked gridlines was used for the repeatable spacing of specimens (Fig 1B). Gridlines were marked for three 3x3 inch pots containing a single plant to be equally spaced in the tray for each image (Fig 1B). Canopeo app developers [17] kept the camera at a uniform distance of 1.5 m from the top of a field grown canopy using a 1.5 m monopod. Following similar methodology, a tripod set at 64 cm (25 in) height was used for taking pictures in a laboratory setting with the smartphone at a repeatable distance from the tray (Fig 1C). A standard square ruler was placed on the outside of the tray. Tripod feet were centered behind the tray, using ruler as a guide for repeatable placement. The Canopeo app was downloaded onto an Android® smartphone. The camera angle was set directly over the tray. Camera settings were set to a default 3 second delayed timer to eliminate camera movement. Imaging occurred in a lab setting with overhead fluorescent lighting.

#### Calibration of the imaging system

Canopeo does not require calibration for use [17], but a visual calibration was performed with solid-colored, green index cards. The image setup was calibrated by capturing images of green, rectangular index cards at two different heights from the camera. The calibration standard was the shape and uniformity of index cards detected as green pixels by Canopeo. Two imaging heights were used because the apparent size of the plant increases as it nears the camera lens proportional to the square of the distance from the camera [18]. Plants growing toward a camera lens may appear to increase in size, causing an overestimation of ground cover. Three plant pots were equally spaced at a repeatable distance on the tray. For the first calibration image, three green index cards were placed on top of each pot at a height of 4 inches from the camera. For the second calibration image, index cards were placed on top of each pot at a height of 8 inches from the camera. Raw images were uploaded to Canopeo MATLAB. The Canopeo software is based on color ratios of red to green (R/G) and blue to green (B/G) and an excess green index. The default parameters [17] used for color ratios in all images were: R/G = 0.95, B/G = 0.95, excess green index = 20. Results showed the first calibration image detected as 8.56% GCF and the second calibration image detected as 11.65% GCF with minimal background noise. This was expected because the closer the object is to the camera lens, the larger the object appears. The shape of each index card was clearly and uniformly captured in each image, indicating Canopeo was calibrated accurately to detect the green light reflected by the green index cards.

Plant inflorescences were trimmed just prior to imaging. This avoids the overestimation of biomass as inflorescences present might touch the camera lens [18]. The Canopeo app measured and estimated the green canopy cover for each genetic line by capturing an image of three plants of the same genotype in the tray. In few cases, only two plants of the same genotype were placed in the tray for imaging. This occurred during senescence when 1–2 plants no longer contained green or yellow pigment and was therefore removed, resulting in an lesser number of replicate specimens for one or two sets, however we have performed multiple experiments for repeatable data and confirmed that they show same biomass measurements. The more advanced senescence is, the more necrotic tissue will be, and less estimate biomass accumulation is digitally captured as green reflected light [31]. The number of technical replicates used in each dataset are made available in the underlying data SAS output tables. Three to four replicate images of each genotype were collected, using each plant only once per developmental stage.

#### Calibration of Canopeo GCF to biomass

Canopeo measurements of GCF were first calibrated with dry biomass weights to estimate biomass accumulation as grams. Plants were imaged at the 6-week developmental stage, immediately followed by manual harvesting, and weighing of the above-ground biomass of the plant. Plant material from each specimen was collected in individual envelopes and placed in an oven to dry at 60°C for seven days. On the seveth day, dried biomass was weighed, and recorded for individual plants. Dry plants were weighed with and without reproductive biomass.

### Experimental setup

After Canopeo calibration of GCF to estimate green biomass as grams, Canopeo estimated green biomass accumulation as grams across six developmental stages using the imaging system (Fig 1) and protocol developed in this study. Collecting biomass accumulation data across the plant life cycle has served to investigate Canopeo’s usefulness for phenotyping visual traits among the three genotypes. Time and cost values were noted as experiments were conducted.

**Fig 2:**
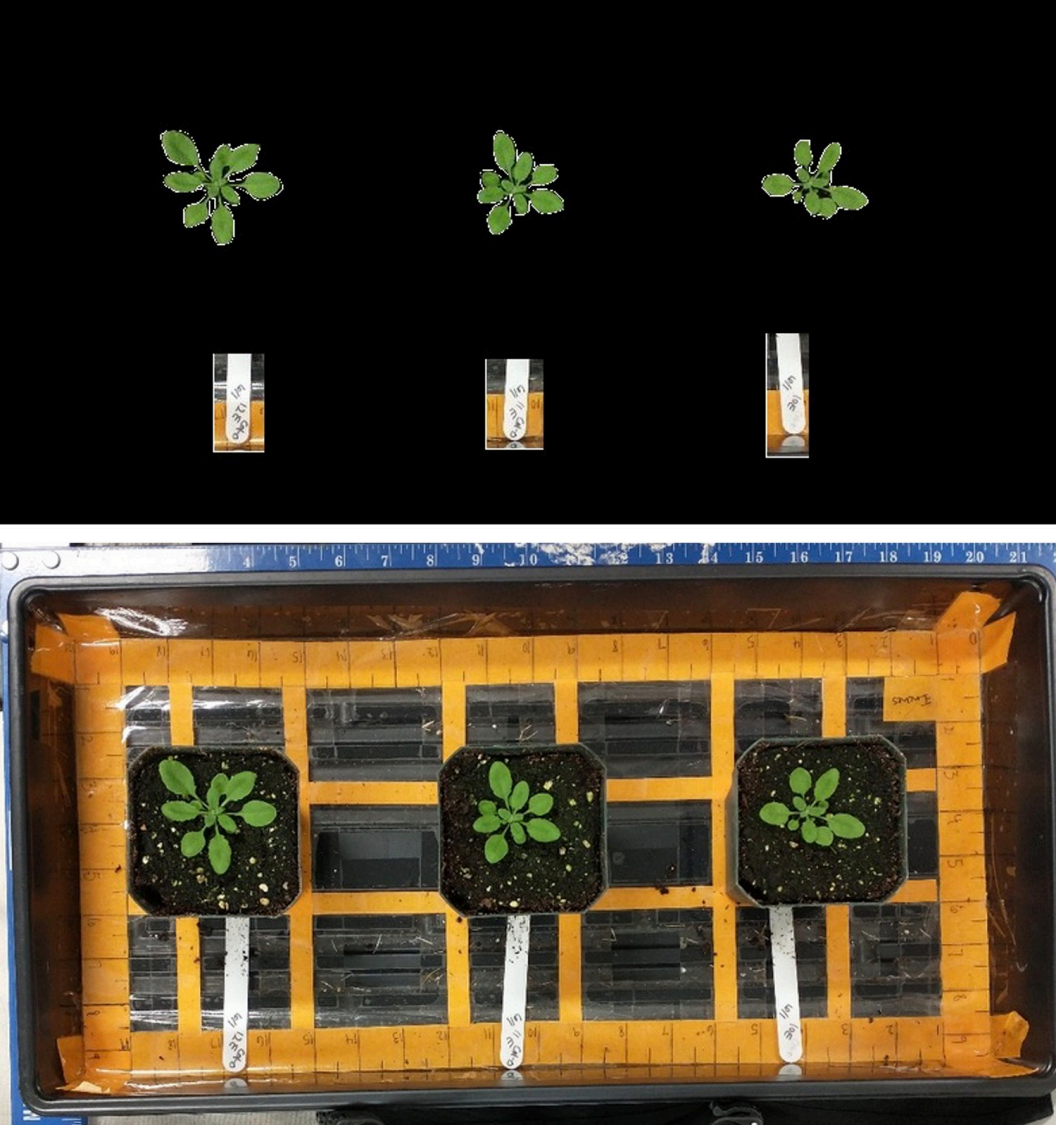
Images from the smartphone were imported into ImageJ software for the purpose of reducing background noise in the images. (Upper panel) Image pre-processing using ImageJ. (Lower panel) raw RGB image imported to ImageJ. The freehand selection tool was used to carefully outline each plant in the image.

Seedling biomass accumulation experiments have served to collect detailed biomass accumulation data during 3-week and 4-week biological stages to compare Canopeo’s built-in noise reduction capabilities with precision background noise reduction. Raw RGB images were manually uploaded and processed in ImageJ software (Version 1.53 a) [39] to reduce background noise (Fig 2) using the lasso tool by carefully outlining the perimeter of each plant by hand. The lasso tool was also used to outline the plant identification markers for each specimen, which did not include any residual green reflected light. Any object not outlined with the lasso tool was blacked out in the RGB image. This preprocessing step in ImageJ resulted in a more accurate estimate of biomass accumulation in Canopeo. Preprocessed images (Fig 2, top panel) were then manually uploaded via batch processing to the desktop version of Canopeo app in MATLAB. Four replicates (n = 4) were used per genotype per experiment. Six independent experimental replicates were used.

Early vigor experiments, which did not undergo reduction of image background noise, served to collect green biomass accumulation data across 3-week, 4-week, and 6-week biological stages. These experiments also served to develop a predictive model for early reproductive biomass partitioning. Four replicates (n = 4) were used per genotype per experiment. Three independent experimental replicates were used.

Senescence onset experiments have served to collect green biomass accumulation data across 8- week, 10-week, and 12-week biological stages. Canopeo was found valuable for monitoring Quinoa senescence in addition to plant canopy expansion [24]. Canopeo detects green light reflected off the plant, but once the plant enters senescence the green pigment in leaves begins to progress gradually from yellow to brown color. This causes Canopeo to detect fewer green pixels in the digital image, thus resulting in a lower estimation of biomass accumulation. This does not indicate a decrease in biomass, rather, it visually indicates the rate at which senescence onsets for each genotype. The more advanced senescence is, the more necrotic tissue will be, lacking green pigment, and less estimate biomass accumulation is digitally captured as green reflective pigment. Three replicates (n = 3) were used per genotype per experiment. Five independent experimental replicates were used. Sample size and replicates in this study exceed the protocol developed by Chung et al. [19] for using Canopeo to phenotype shorghum cultivars in pots, where three replicates with five plants per replicate were used and where images were taken from the third week, when 3–4 leaves had emerged, at one week intervals for five weeks.

**Fig 3:**
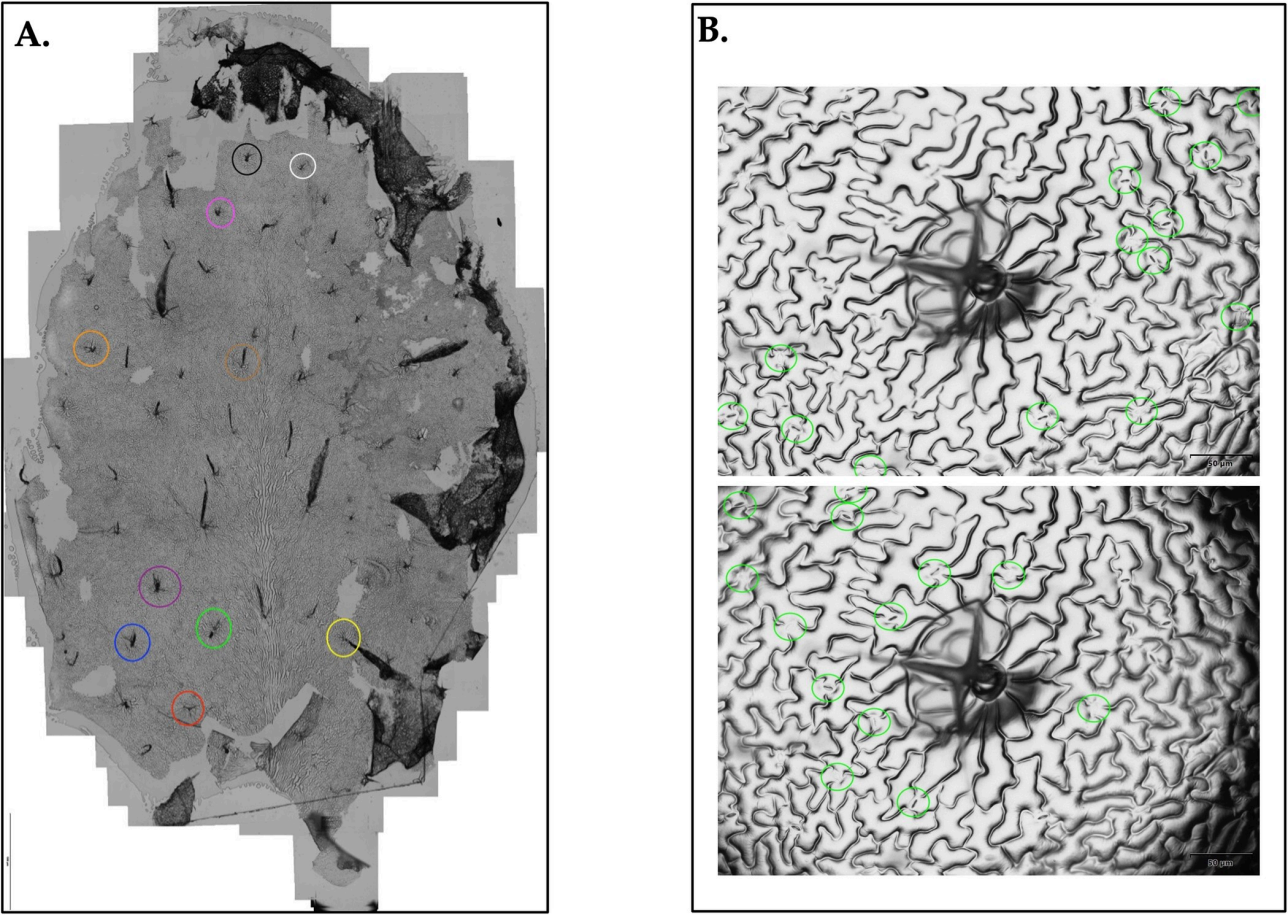
Sampling for stomatal density. (A) Col-0 whole leaf imprint shows five trichomes selected in each distal and proximal region for stomatal density analysis. (B) A single trichome is centered in the microscope field of view and 27 stomata are manually counted surrounding the trichome.

Stomatal density experiments were used for estimating the number of stomata surrounding trichome epidermal cells for each genotype. First, clear nail varnish was used to take stomatal imprints of the adaxial surface of 5^th^ and 6^th^ leaves of each Arabidopsis rosette using the described protocol [40]. Three leaves (n = 3) were imprinted from three individual plants for each genotype in each experiment. Six independent experimental replicates were used. In Col-0, stomatal density appears as a gradient along the length of the leaf from proximal to distal end [41]; therefore, adaxial stomatal density was sampled according to leaf region (proximal and distal). Next, five trichomes were selected at random in each proximal and distal region of sampling (Fig 3A). A single trichome epidermal cell was centered in the microscope (Olympus DP80) field of view using the “target” function and using uniform magnification. Stomatal density was sampled by manually counting the stomata surrounding each individual trichome in the field of view (Fig 3B). The five sampled values were averaged together, respective of region, to obtain a proximal and distal stomatal density value for each leaf. Thus, a total of six averaged stomatal density values per genotype (two regional values from each of three leaves) were obtained per experimental replicate. Finally, an average stomatal density value was obtained for each leaf by averaging together the distal and proximal values of each leaf. For plants grown in a low-light environment, stomatal density was measured from plants in the 8-week developmental stage. For plants grown in a high-light environment, stomatal density was measured from plants in the 6- week and 8-week developmental stages.

Stomatal frequency experiments were used for estimating the number of stomata per leaf area for each genotype. Leaves from the low light intensity environment were imaged with a microscope at the 8-week and 10-week developmental stages using two independent experimental replicates. Leaf area was measured with ImageJ. Complementary samples of distal and proximal stomatal density were also measured at the 8-week and 10-week developmental stages.

### Statistical analyses

Collecting green biomass accumulation data across the plant life cycle have served to investigate genotype by environment interactions that could be used for phenotyping biomass related traits, including early vigor and onset of senescence. Each developmental stage constituted an individual dataset, respective to each independent experiment. Mean separation analyses using SAS® Glimmix were used for measuring interactions among genotype, estimate biomass accumulation, and developmental stage. Statistically significant data outputs were grouped alphabetically according to LS-means (p<0.05). Simple linear regressions were performed using Micrososft Excel® The Regression Analysis in the data analysis Tool-Pack was used to evaluate both the coefficient of determination (r^2^) and the p-value for the F-test for overall significance (Significance F), (p<0.05).

The measurement of GCF as a percentage can be used directly for monitoring relative growth rates [18]; therefore, Canopeo measurements of estimated green canopy cover in this study were first correlated with dry biomass to obtain a y-intercept equation (S1 Fig). This equation was subsequently used to convert Canopeo GCF into estimate biomass accumulation as grams. Calibration of Canopeo biomass accumulation estimates with dry biomass as grams was performed by correlating data to obtain an r^2^ value. This served to validate the accuracy of Canopeo measurements in the lab by determining the model’s goodness of fit.

Data from early vigor experiments and senescence onset experiments were used to compare biomass accumulation estimates between early and late developmental stages. Data from the 12-week developmental stage were compared to 3-week and 4-week data. Three independent experimental replicates were used. The exact same plants were not able to be measured with Canopeo through all three developmental stages because destructive biomass measurements were taken at the 6-week developmental stage.

A predictive model for early reproductive biomass partitioning was developed using a regression analysis. Estimate biomass accumulation values (excluding reproductive biomass) measured at 3-week and 4-week developmental stages were plotted as linear regressions against total dry biomass weights (including reproductive biomass) measured at the 6-week developmental stage using the same plants. For each genotype, dry weights were individually regressed against the 3-week and 4-week biomass accumulation estimates, respective of each plant specimen within each experimental replicate. Three experimental replicates were used for the predictive model analysis.

The dependence of biomass production on stomatal density was tested in plants with FKF1 mutation. A linear regression of complementary measurements from stomatal density experiments and biomass accumulation experiments served to investigate whether biomass production is dependent upon (i.e., is a function of) stomatal density in plants with FKF1 mutation. Reasoning for this is stomatal density is a component of stomatal conductance. Stomatal conductance, in turn, influences rate of CO_2_ fixation and thus biomass accumulation. Complementary measurements were correlated from the same plants within each independent experiment. Regression analyses were performed for developmental stages at 6-, 8-, 10-, and 12-weeks old.

To further elucidate whether biomass production is dependent upon stomatal density, stomatal frequency was estimated- which is the number of stomata per leaf area. First, stomatal density was calculated per specimen by summing distal and proximal stomatal density values for each developmental stage. Where stomatal density was sampled across two developmental stages for a single specimen, distal and proximal stomatal density values were first added by developmental stage and then the two resulting values were averaged together. Next, leaf area was calculated in mm^2^ per specimen for each developmental stage. Where leaf area was sampled across two developmental stages for a single specimen, the sum of distal and proximal stomatal density per stage was multiplied by leaf area per stage. The resulting two values for each developmental stage were then averaged to obtain x-axis values of stomata per leaf. Where leaf area was sampled for just one developmental stage for a single specimen, y-axis values were multiplied by leaf area to obtain x-axis values. A linear regression analysis served to estimate stomatal frequency for each genotype.

## Results

### Analysis of time and cost

Imaging setup, data capture, and setup disassembly was performed in 30 minutes or less. For a single experiment, image upload to Canopeo took 15 minutes, including time to add a label to each image in the app. Data could easily be archived to Microsoft Excel ®. Statistical analyses of Canopeo data using SAS ® GLIMMIX took less than 30 minutes, respective to each independent experiment. The imaging system was applied with a budget of under $100, excluding smartphone model- which is up to personal preference.

Detecting seedling biomass accumulation estimates required additional processing steps after image acquisition (Fig 2) to reduce background noise. Manually processing a single dataset (12 photos) through ImageJ took approximately 1.5 hours. This process was applied to six replicate experiments imaged across two developmental stages, adding up to a total of 18 hours.

### Calibration of dry and digital biomass

**Fig 4:**
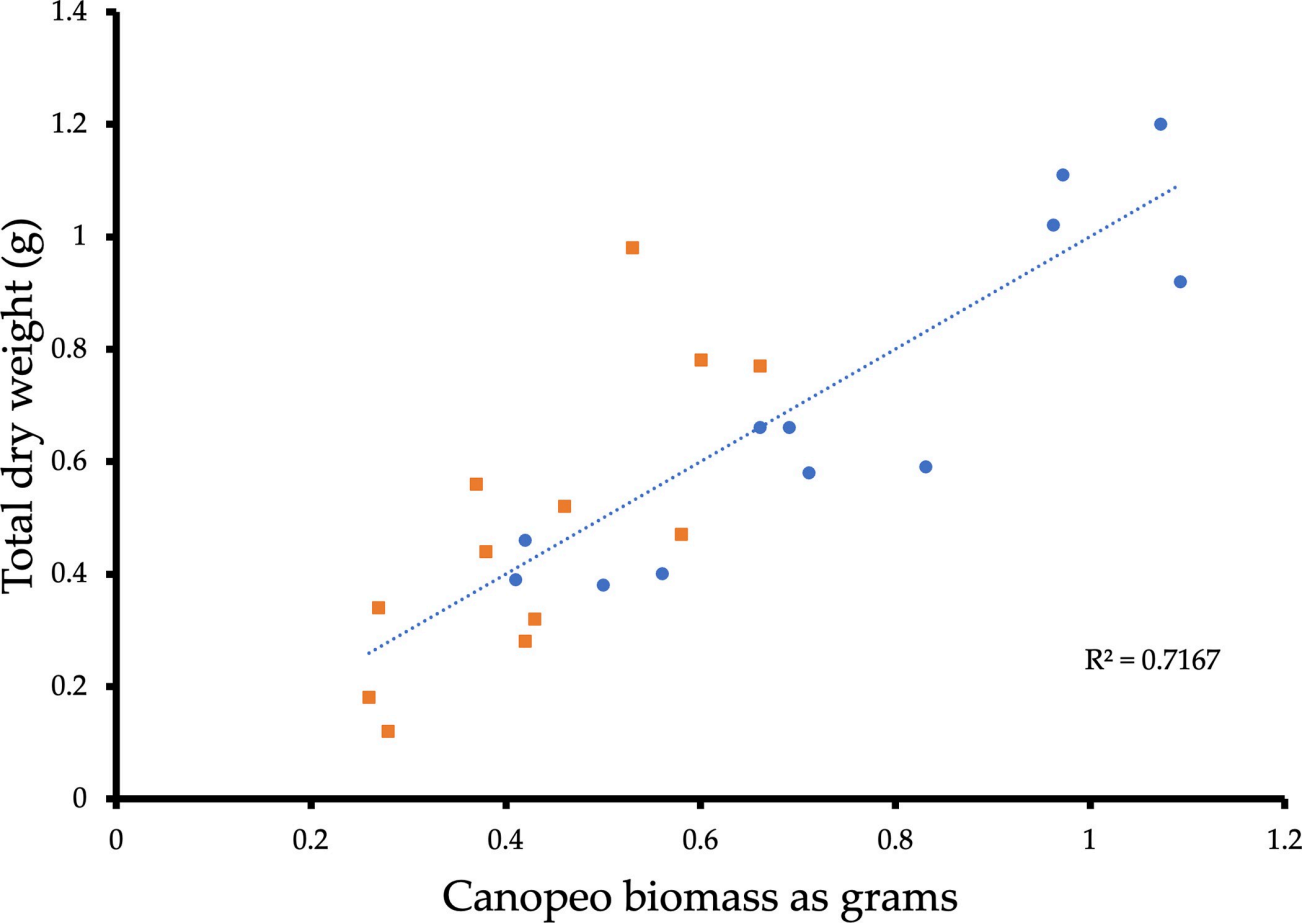
Calibration of Canopeo measurements in grams. Canopeo biomass was calibrated using dry plant weights. Blue dots and orange squares represent different experimental replicates.

Canopeo measurements of estimated biomass accumulation as grams were found to be consistent with the traditional method of destructive plant biomass measurements (r^2^ = 0.72), demonstrating dry plant biomass is directly related to biomass accumulation estimates as grams measured by Canopeo in a lab setting (Fig 4).

### Phenotyping biomass in grams

Canopeo estimated biomass accumulation as grams for three Arabidopsis genotypes (Col-0, *fkf1-t*, FKF1-OE) across six developmental stages. Seedling biomass accumulation analysis (Table 1) showed *fkf1-t* to have an above average seedling vigor in four out of seven experiments, along with a later onset of senescence compared to the other genotypes. LS-means tables for each experimental replicate by developmental stage interaction demonstrated six out of twelve tables had significant ranking of *fkf1-t* based on numerical order and rank alone, excluding alphabetical groupings (S1 Table). Four out of twelve tables demonstrated the FKF1-OE genotype had significant order and rank based on numerical value alone. One out of twelve tables demonstrated *fkf1-t* and FKF1-OE were tied in rank due to identical numerical values for both genotypes. Similarly, one out of twelve tables demonstrated *fkf1-t* and Col-0 were tied in rank due to identical numerical values for both genotypes. Overall, data collected at the 4-week developmental stage revealed greater variation among biomass accumulation rates compared to 3-week developmental stage data.

**Table 1:** Estimate biomass accumulation as grams by week in seven experiments comparing *fkf1-t*, Col-0, and FKF1-OE plants with twelve biological replicates per experiment.

Experiment	Line	Week 3	Week 4	Senescence (Week 12)
		Estimate biomass accumulation (g)		
1	*fkf1-t*	0.26 (a)	0.35 (a)	
	Col-0	0.25 (a)	0.34 (a)	
	FKF1-OE	0.26 (a)	0.35 (a)	
2	*fkf1-t*	0.24 (a)	0.26 (a)	
	Col-0	0.24 (a)	0.25 (a)	
	FKF1-OE	0.24 (a)	0.26 (a)	
3	*fkf1-t*	0.28 (a)*	0.48 (a)*	
	Col-0	0.26 (b)	0.40 (b)	
	FKF1-OE	0.27 (ab)*	0.43 (ab)*	
4	*fkf1-t*	0.25 (a)	0.36 (a)	
	Col-0	0.25 (a)	0.35 (a)	
	FKF1-OE	0.25 (a)	0.34 (a)	
5	*fkf1-t*	0.32 (ab)*	0.57 (a)	
	Col-0	0.30 (b)	0.53 (a)	
	FKF1-OE	0.33 (a)*	0.53 (a)	
6	*fkf1-t*	0.25 (a)	0.46 (a)*	
	Col-0	0.25 (a)	0.44 (ab)	
	FKF1-OE	0.25 (a)	0.40 (b)*	
7	*fkf1-t*	0.27 (a)	0.46 (a)*	0.47 (a)*
	Col-0	0.27 (a)	0.44 (b)	0.30 (b)
	FKF1-OE	0.28 (a)	0.43 (b)	0.28 (b)

†Means with the same letter within an experiment and week are not significantly different from each other (P_crit_ = 0.05).

**Fig 5:**
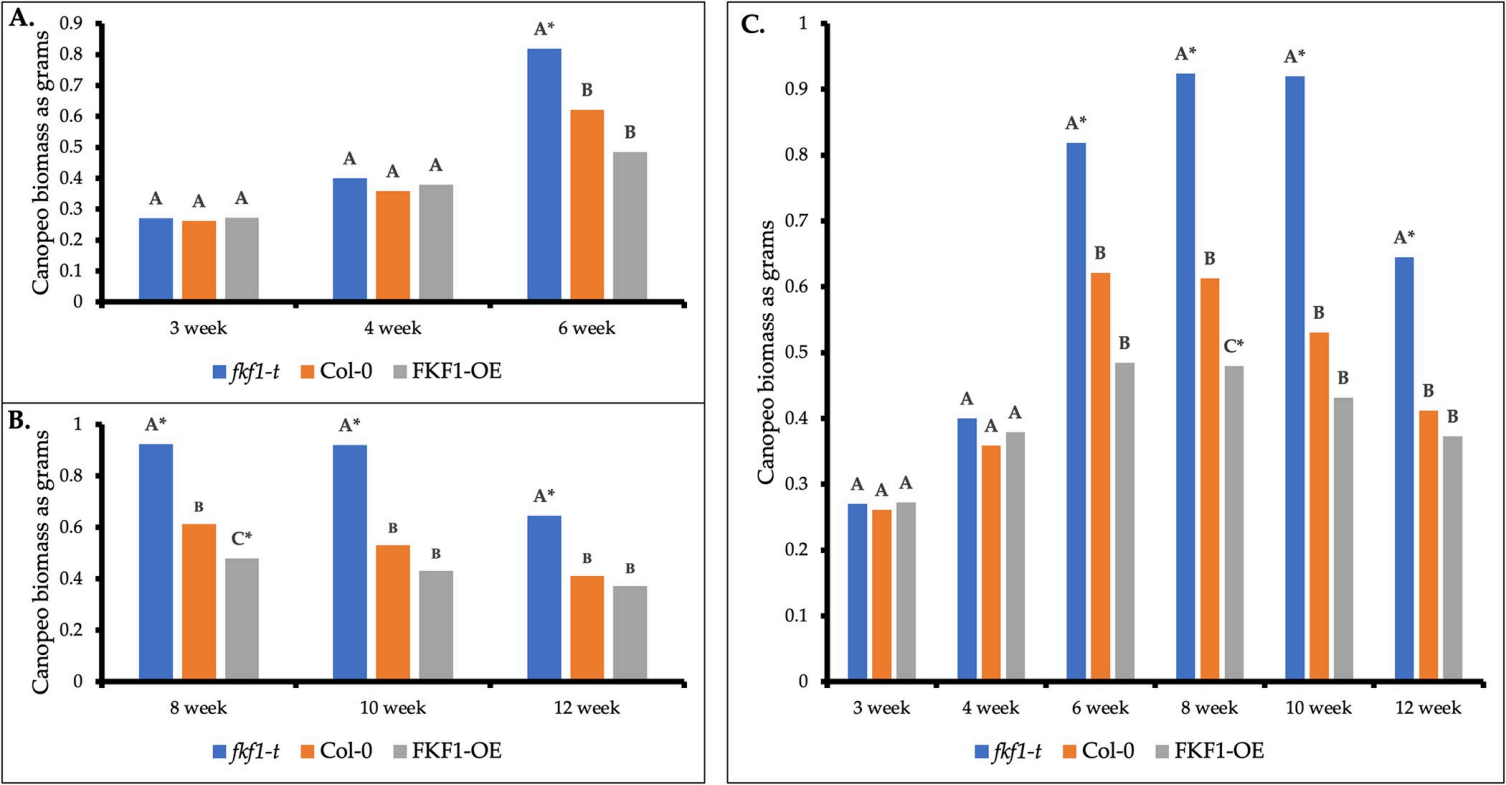
Green biomass by week: *Fkf1-t*, Col-0, FKF1-OE. (A) Biomass accumulation of *fkf1-t* was consistently higher at the 6-week developmental stage compared to Col-0. (B) Biomass accumulation of *fkf1-t* remained consistently higher across 8-, 10-, and 12-week developmental stages compared to Col-0. Relative biomass of FKF1-OE was statistically lower than Col-0 at the 8-week developmental stage. By week 12, overall lower relative biomass measurements indicate all plants are in senescence, but the statistically significant grouping of *fkf1-t* suggests that this genotype experiences senescence at a slower rate. Statistically significant difference between Col-0 and any other *Arabidopsis* line is labeled with * (p < 0.05). (C) Detecting biomass accumulation with Canopeo across six developmental stages provides insights on rate of vegetative biomass partitioning and rate of senescence across the plant life cycle. Statistical analyses were performed using SAS GLIMMIX. Means with same letter within same week are not significantly different. Statistically significant difference between Col-0 and any other Arabidopsis line is labeled with * (p<0.05).

The method statistically differentiated between the estimate green biomass accumulation of *fkf1-t* (0.82 grams) and Col-0 (0.62 grams) beginning at the 6-week developmental stage (Fig 5A). Biomass accumulation of all three genotypes, *fkf1-t* (0.92 grams), Col-0 (0.62 grams), and FKF1-OE (0.48 grams), began differentiating at the 8-week-developmental stage (Fig 5B). The green biomass accumulation of *fkf1-t* remained consistently greater across 6-week, 8-week, 10-week, and 12-week developmental stages relative to Col-0 (Fig 5C).

**Fig 6:**
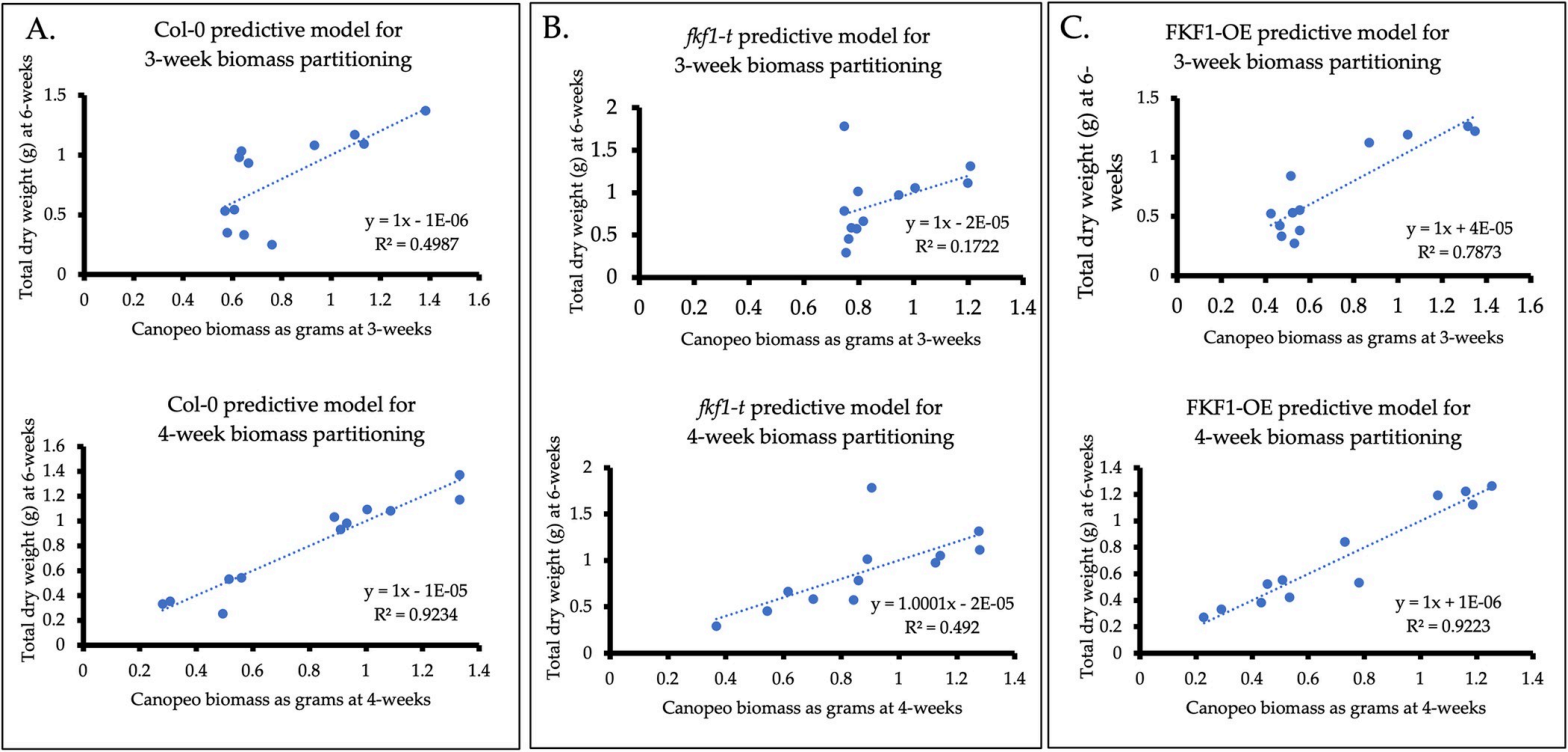
Regression analysis of early-development Canopeo measurements with mid-development dry biomass as a predictive model for early reproductive biomass partitioning. FKF1-OE biomass accumulation was closely related to dry biomass measured at the 6-week stage, highlighting FKF1-OE’s shorter lifecycle and reduced vegetative biomass partitioning. *fkf1-t* biomass accumulation was least related to dry biomass measured at the 6-week stage, thereby demonstrating exponential growth. (A) Col-0 biomass partitioning at the 3-week and 4-week developmental stages. (B) *fkf1-t* biomass partitioning at the 3-week and 4-week developmental stages. (C) FKF1-OE biomass partitioning at the 3-week and 4-week developmental stages.

A predictive model for early reproductive biomass partitioning (Fig 6) was developed. For FKF1-OE (Fig 6C), both linear relationships of 3-week (r^2^ = 0.79) and 4-week (r^2^ = 0.92) Canopeo measurements with 6-week dry biomass were stronger than both linear relationships of Col-0 (r^2^ = 0.50 and 0.92), (Fig 6A), and *fkf1-t* (r^2^ = 0.17 and 0.49) (Fig 6B).

### Measuring onset of senescence

Estimate green biomass accumulation as grams slightly decreased between 6-week and 8-week developmental stages for both Col-0 (from 0.621 to 0.613 grams) and FKF1-OE (from 0.484 to 0.479 grams), (Fig 5C). *fkf1-t* estimate green biomass accumulation slightly decreased between 8-week and 10-week (from 0.924 to 0.920 grams) developmental stages. At the 12-week developmental stage, *fkf1-t*’s estimate green biomass (0.65 grams) was greater than the estimate green biomass of both Col-0 (0.41 grams) and FKF1-OE (0.37 grams).

### Associating biomass, stomatal density, and leaf area

**Fig 7:**
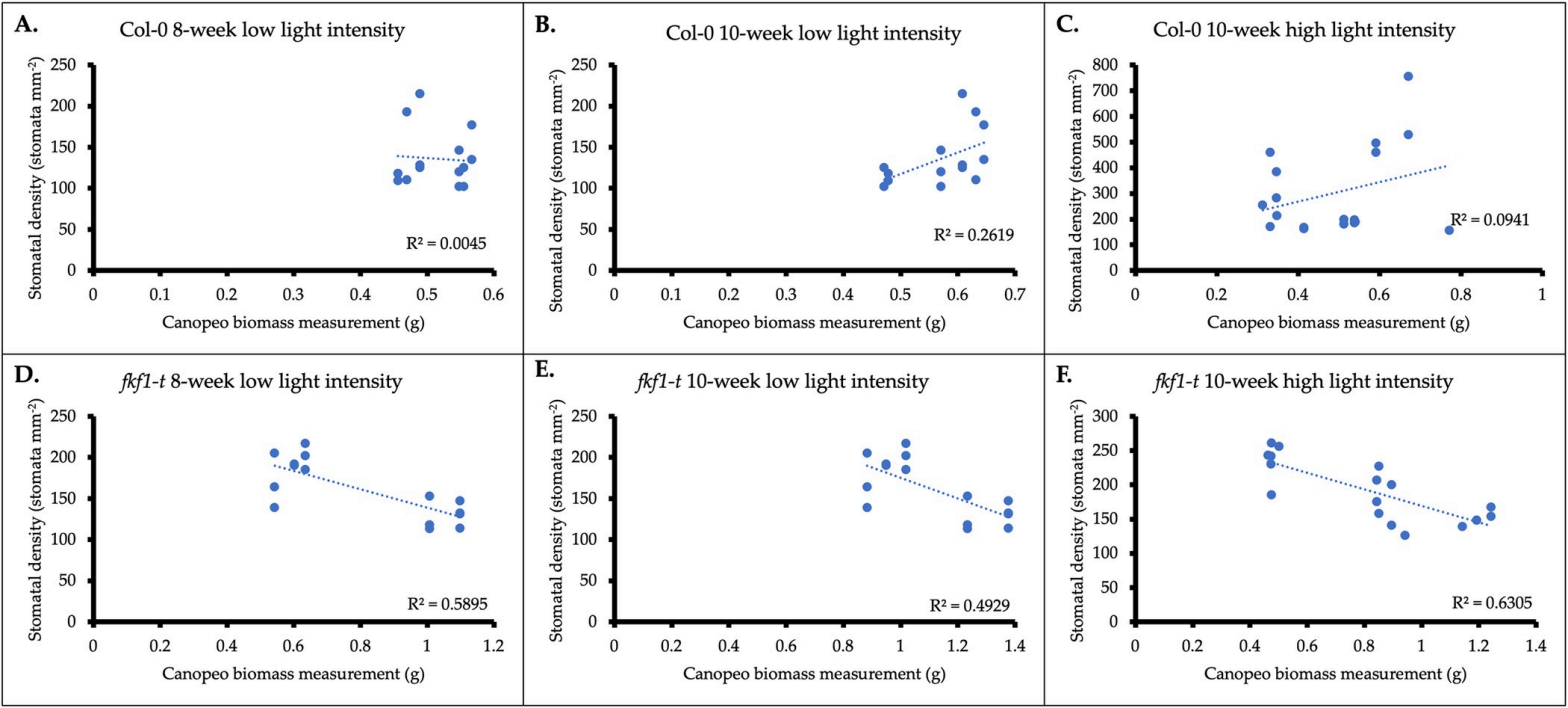
Relationship of Canopeo based biomass with Arabidopsis thaliana stomatal density in *fkf1-t*. *fkf1-t* plants grown under low light intensity and high light intensity demonstrated increased stomatal density with lower biomass. (A) Relationship of stomatal density and biomass of Col-0 at the 8-week developmental stage grown under low light intensity conditions. (B) Relationship of stomatal density and biomass of Col-0 at the 10-week developmental stage grown under low light intensity conditions. (C) Relationship of stomatal density and biomass of Col-0 at the 10-week developmental stage grown under high light intensity conditions. (D) Relationship of stomatal density and biomass of *fkf1-t* at the 8-week developmental stage grown under low light intensity conditions. (E) Relationship of stomatal density and biomass of *fkf1-t* at the 10-week developmental stage grown under low light intensity conditions. (F) Relationship of stomatal density and biomass of *fkf1-t* at the 10-week developmental stage grown under high light intensity conditions.

The relationship of Canopeo based biomass with stomatal density in *fkf1-t* (Fig 7) was negative and linear compared to Col-0, demonstrating increased stomatal density with lower biomass in both low-light (r^2^ = 0.59 and 0.49) and high-light (r^2^ = 0.63) environments. Further, the p-values associated with the overall F-test statistics indicate that the regression models are statistically significant for the *fkf1-t* genotype (S2 Table), (p<0.05). *fkf1-t* and Col-0 genotypes grown under high-light intensity demonstrated greater stomatal density compared to low-light intensity experiments, respective of developmental stage. However, *fkf1-t* grown under high-light intensity revealed less stomatal density compared to Col-0 grown under the same conditions.

**Fig 8:**
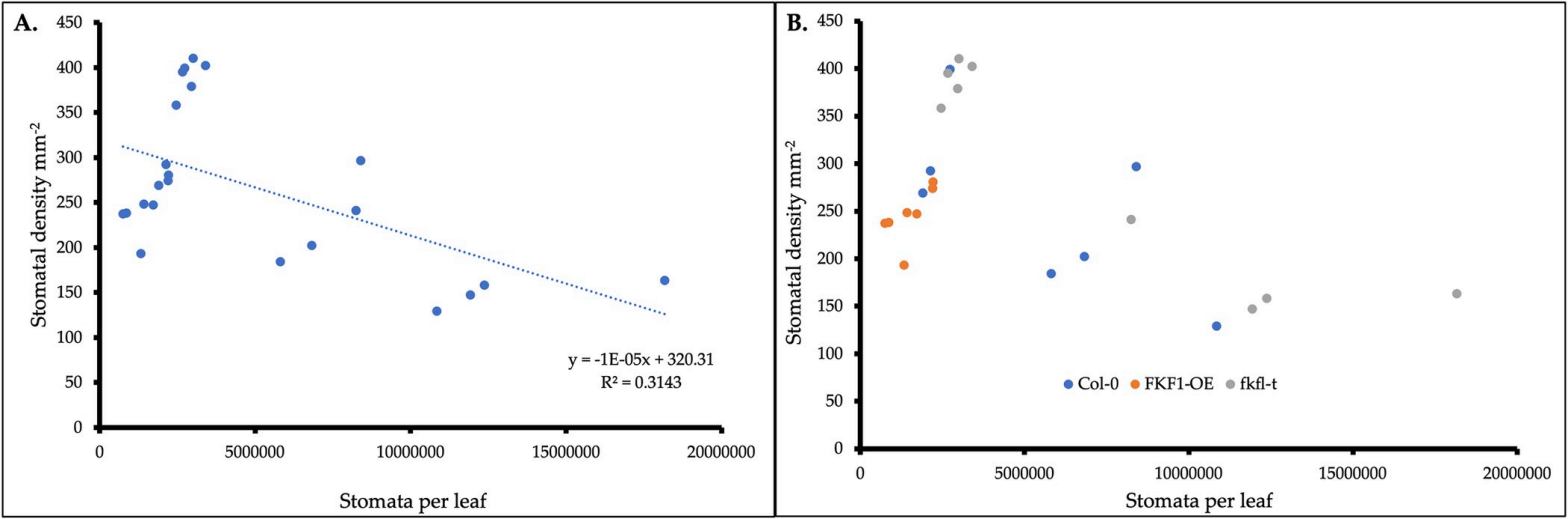
Estimation of stomatal frequency. **(**A) plants with lower stomatal density surrounding trichomes tended to have a greater stomatal frequency when grown under lower light intensity. (B) FKF1-OE demonstrated the least variation in stomatal frequency.

To further elucidate whether biomass accumulation is dependent upon stomatal density, stomatal frequency was estimated by determining the number of stomata per leaf area (Fig 8). A linear regression analysis was performed between estimated total stomata per leaf and the average stomatal density sampled per specimen. Results yielded a negative correlation between stomata per leaf and stomatal density. The p-value associated with the overall F-test statistic indicates the regression model is statistically significant (S3 Table), (p<0.05). All sampled FKF1-OE specimens were clumped together at the far-left side of the scatter plot, suggesting that this genotype experienced the least amount of variation for stomatal frequency trait.

## Discussion

### Cost-efficient lab phenotyping with Canopeo

Canopeo was adapted for lab use (Fig 1) with a budget of under $100, excluding the smartphone. Recording 12 pictures and processing in the Canopeo app took 15 minutes. In contrast, manual biomass measurements took nearly twice the amount of time whencompared to image-based measurements, excluding the time for oven drying. This comparison was not set up with replicated statistical analysis, but rather to inform users of the potential time requirements for collecting such data. These findings are in agreement with a previous report that the Canopeo phone app requires one minute per image for recording and processing, and manual measurements are more time consuming [33].

Next, Canopeo-MATLAB was tested for its cost efficiency in phenotyping seedling biomass accumulation traits (Table 1) via batch processing of images. In this study, ImageJ and Canopeo-MATLAB were used in tandem to process image-based datasets. Less than 20 hours were spent manually reducing green-colored background noise of images with ImageJ (Fig 2) prior to batch processing via Canopeo-MATLAB. A previous study comparing ImageJ and Canopeo-MATLAB detection of GCF found that they are both accurate and low-labor choices for batch processing of large numbers of images [33]. However, this previous study used RGB-based threshold settings in ImageJ as opposed to manual noise reduction methods.

### Phenotyping biomass with Canopeo

The FKF1-OE genotype’s reported early flowering time [38] coupled with less green biomass accumulation across its lifecycle (Fig 5C) and average earlier onset of senescence (Table 1) illustrates a more determinative growth habit where plant resources are primarily allocated for seed production. The strong relationship between early green biomass accumulation and dry biomass measured at the 6-week stage (Fig 5C) further highlights FKF1-OE’s shorter lifecycle and reduced vegetative biomass partitioning. The *fkf1-t* genotype can be characterized in part by late flowering time under long-day photoperiod conditions [37]. Late flowering phenotypes have more time to accumulate biomass vegetatively before entering senescence, consistent with greater green biomass accumulation across its lifecycle (Fig 5C) and later onset of senescence (Fig 5C and Table 1). These findings illustrate an aggressive vegetative growth habit with resource partitioning favoring leaf biomass accumulation. The weak relationship between early green biomass accumulation and dry biomass measured at the 6-week stage (Fig 5B) further highlights *fkf1-t*’s exponential growth compared to the other genotypes. *fkf1-t* has a late flowering time phenotype and longer vegetative phase compared to the Col-0 and FKF1-OE. Therefore, it should follow that *fkf1-t* would grow at a slower rate during early developmental stages. However, *fkf1-t* demonstrated early vigor traits (Table 1), which remains unexplained from a resource partitioning perspective. Findings of this study may also suggest *fkf1-t* is less sensitive to environmental stressors; however, additional experiments are needed to confirm this hypothesis.

Calibration between dry vegetative biomass and green canopy cover measured with Canopeo served to phenotype biomass accumulation as grams. Canopeo measurements of estimated biomass accumulation as grams were linearly related to dry biomass measurements (r^2^ = 0.72), indicating a 1:1 relationship with ground cover for the method (Fig 4). These results agree with findings that ground cover measurements have the potential to be accurate estimators of crop growth because the images are useful in determining vegetation indices and canopy measurements were closely related to crop growth and radiation capture (r^2^ ranging from 0.62 to 0.99) [17–20,24,32–35,42,43]. Results also agree with field-based studies reporting the high accuracy of Canopeo for estimating biomass of winter cereal cover crops (r^2^ = 0.52) [25], phenotyping sorghum biomass (r^2^ = 0.88 to 0.96) [19], and phenotyping shoot biomass of lentil (r^2^ = 0.66) [20].

Imaged-based measurements of estimate green biomass accumulation identified biomass accumulation traits and senescence onset traits across the plant lifecycle. *fkf1-t* was identified with greater seedling biomass accumulation vigor compared to Col-0 at 3- and 4-week developmental stages (Table 1) when noise reduction of images in ImageJ occurred before Canopeo processing. Without precision noise reduction of images, the method has reduced efficiency for detecting early vigor biomass accumulation traits, thereby differentiating biomass accumulation rate of *fkf1-t* from Col-0 at a later developmental stage (Fig 5A).

Canopeo’s performance for phenotyping biomass of matured crop canopy was first reported by Ashapure *etal* in 2019 [32]. This field-based study on cotton found Canopeo inefficient at identifying matured crop canopy because the canopy started to change color from green to yellow and eventually to brown. However, this present study interpreted decreasing green estimate biomass accumulation values as a visual marker for onset of senescence [24,44,45]. Results indicate that Col-0 and FKF1-OE showed senescence between 6 and 8 weeks (Fig 5C), while *fkf1-t* enters senescence at a slower rate between 8 and 10 weeks. By 10 and 12 weeks, all genotypes reflected decreased estimate green biomass accumulation compared to previous weeks. This was expected because it indicates all genotypes progressing to senescence. At the 12-week developmental stage, the estimate green biomass accumulation of *fkf1-t* was more than double the green biomass values of the other genotypes. These findings support that the *fkf1-t* genotype experiences senescence at a slower rate. On average, FKF1-OE appeared to enter senescence earlier than Col-0 (Table 1). To the best of our knowledge, only one prior study has confirmed the usefulness of Canopeo for detecting onset of senescence in a field setting [24]. However, it is not well understood how senescence timing was controlled by plants [46]. While plant senescence is due in part to the aging process, it does not necessarily mean plants have to reach a certain age to senesce.

Finally, a predictive model for early reproductive biomass partitioning was developed. The strength of linear relationships of the predictive model decreased as flowering-time phenotype became increasingly delayed: FKF1-OE r^2^ = 0.79 and 0.92; Col-0 r^2^ = 0.50 and 0.92; *fkf1-t* r^2^ = 0.17 and 0.49, respectively (Fig 6). These results were expected because they coincide with reports that FKF1-OE is an early flowering phenotype with a small leaf rosette size [38] compared to the wild type (Col-0) or *fkf1-t* mutant. Col-0 flowering-time phenotype is intermediate between the two mutants, while *fkf1-t* has a late flowering phenotype [37].

In the present study, the Canopeo app was applied as a RGB close-range remote sensing tool in a laboratory setting. Advantages of RGB sensors include low-cost, simplicity, accessibility, and automation of data acquisition which saves time and cost relative to manual field monitoring. Multi-spectral and hyperspectral image sensors can provide more detailed spectral information. Hyperspectral image sensors are more accurate than multispectral or single-band image sensors. However, obstacles such as price of the sensors, image processing, and image analysis have limited the accessibility of both multispectral and hyperspectral sensors as a phenotyping tool [7,12]. Smartphones can be adapted for the purposes of multispectral and hyperspectral imaging [47], but a readily available smartphone application is currently not available for download and use. Depth image sensors and LiDAR can both be used for 3-D reconstruction of plants to estimate plant traits [13,14], thus offering more detailed data for phenotyping both morphometric and physiological parameters. However, like multispectral and hyperspectral sensors, a readily available smartphone application for these types of sensors is not available for download thereby limiting the technology’s accessibility and simplicity of use. Multispectral, hyperspectral, depth image sensors, and LiDAR sensors all offer more detailed data acquisition compared to RGB methods. However, RGB remote sensors offer the greatest level of accessibility and simplicity of use with readily available smartphone applications available for public download. Further, the accuracy of our results (r^2^ = 0.72) agree with field-based studies reporting the high accuracy of Canopeo for estimating biomass of winter cereal cover crops (r^2^ = 0.52) [25], phenotyping sorghum biomass (r^2^ = 0.88 to 0.96) [19], and phenotyping shoot biomass of lentil (r^2^ = 0.66) [20]. In addition, Table 2 provides a comparison of accuracy of different visible light imaging techniques for biomass estimation in plant phenotype application. The accuracy of our approach outperformed the accuracy of similar systems that used Canopeo to measure shoot biomass of lentil and winter cereal cover crops in the field (Table 2 and Fig 4). Further, the accuracy of our approach performed nearly the same as the system that used Canopeo to measure biomass production of Old World Bluestem in the field. This comparable performance between systems emphasizes the beneficial use of Canopeo in a laboratory setting for assessing phenotype parameters related to growth and vigor. However, using the Adobe Photoshop system to measure growth rate of lettuce in a laboratory setting yielded higher accuracy than all the Canopeo systems listed in Table 2. Further, the accuracy of our approach performed nearly the same as the system that used Canopeo to measure biomass production of Old World Bluestem in the field. This comparable performance between systems emphasizes the beneficial use of Canopeo in a laboratory setting for assessing phenotype parameters related to growth and vigor. Taken together, this highlights RGB close-range remote sensing as a good compromise between accessibility, end-user simplicity, affordability, and accuracy.

**Table 2:** A comparison of accuracy of different visible light imaging techniques for biomass estimation in plant phenotype application [18–20,24,25,32–34,48].

Imaging technique	Sensor	Software	Sensor estimated parameter	Other measured parameter used for correlation	Accuracy	Phenotype parameters	Example of species	Imaging environment
Visible light imaging (proposed approach)	RGB channel smartphone camera	Canopeo app; ImageJ	Above-ground green biomass as grams	Above-ground biomass	r^2^ = 0.72	Early vigor, senescence onset, lifecycle biomass accumulation	Arabidopsis	Laboratory
Visible light imaging	RGB channel smartphone camera	Canopeo app	Percentage of green canopy color	Plant height and node height	r^2^ = 0.88 to 0.96 [19]	Biomass accumulation	Sorghum	Greenhouse
Visible light imaging	RGB channel smartphone camera	Canopeo app	Percentage of green canopy color	Shoot biomass	r^2^ = 0.66 [20]	Shoot biomass	Lentil	Field
Visible light imaging	RGB channel smartphone camera	Canopeo app	Percentage of green canopy color	Root biomass, shoot biomass, nutrient status	r^2^ = 0.52 [25]	Shoot biomass	Winter cereal cover crops	Field
Visible light imaging	RGB channel digital camera	Adobe Photoshop	Percent ground cover	Percent PAR absorption and daily carbon gain	r^2^ = 0.99 [18]	Growth rate	Lettuce	Laboratory
Visible light imaging	RGB channel smartphone camera	Canopeo app	Percentage of green canopy color	Relative chlorophyll content measured with SPAD-502 meter	r^2^ = 0.62–0.87 [24]	Crop biomass	Quinoa	Field
Visible and near-infrared light imaging	RGB channel and multispectral unmanned aerial vehicles	Agisoft Photoscan Pro and SlantView	Green canopy cover	NDVI	r^2^ = 0.98 and 0.97 [32]	Canopy cover modelling	Cotton	Field
Visible light imaging	RGB channel digital camera	Canopeo app and ImageJ	Percent ground cover	Percent PARI (photosynthetically active radiation interception)	r^2^ = 0.72 (ImageJ) and 0.74 (Canopeo) [33]	Biomass production	Old World Bluestem	Field
Visible light imaging	RGB channel smartphone camera and a line quantum sensor	Canopeo app	Percentage of green canopy color	Light interception measured with line quantum sensor	r^2^ = 0.94 (image-based) and 0.92 (video-based) [34]	Canopy cover expansion	Soybean	Field
Visible light imaging	RGB channel digital camera and smartphone Camera	Canopeo app and ImageJ	Percentage of green canopy color	GCF parameter from Canopeo and ImageJ correlated with each other	r^2^ = 0.91 (no colorants applied) [48]	Green coverage of living turfgrasses	Bermudagrass	Field

### Phenotyping stomatal architecture with Canopeo

The secondary purpose for using Canopeo-derived measurements was to guide hypotheses for additional roles of the FKF1 protein. In 2019, the Mendu lab addressed the question of whether delayed flowering is the reason behind increased cellulose content and biomass of *fkf1-t* [37]. Results of the Mendu Lab indicate a specific role of FKF1 in negatively regulating cellulose biosynthesis, and subsequentially biomass. This presents a unique opportunity to test the method described herein for application of reverse genetic approach in phenotyping morphological traits. Understanding the function of the FKF1 gene respective to each genotype is essential for understanding the subsequent phenotype expressed for each mutant. Here, the physiological parameter of estimated green biomass accumulation is analyzed with the morphological parameter of estimated stomatal density to accomplish morphological phenotyping of leaf traits. Trichomes are believed to influence the leaf boundary layer [49,50], which would have consequences on stomatal conductance and rate of CO_2_ fixation in plant biomass. With this in mind, stomatal density was sampled in the area surrounding leaf trichomes.

*fkf1-t* plants with increased stomatal density tended to have lower biomass, while plants with decreased stomatal density tended to have higher biomass, (Fig 7). These findings agree with Cheng-Bin Xiang’s [51], where biomass was increased in mutants and transgenic plants with reduced stomatal density. Further, low light intensity treatments on average had lower stomatal density compared to high light intensity treatments, consistent with findings that high light intensity facilitates increased stomatal density [51]. The clustering of data points demonstrated by the *fkf1-t* graphs (Fig 7, lower panels) may be explained by the nature of the T-DNA insertion mutation. Random integration of T-DNA into the host genome (*fkf1-t*) could affect upstream and/or downstream regulatory genes that play a role in co-influencing biomass accumulation and stomatal density. This may explain the clustering of *fkf1-t* plants into groups (i.e. low stomatal density plants with greater biomass and high stomatal density plants with less biomass). Nontheless, statistical analyses validate the model for demsontrating the relationship between stomatal density and biomass accumulation of the *fkf1-t* genotype. Plants with lower stomatal density tended to have a greater stomatal frequency (Fig 8) when grown under low-light intensity. The negative correlation was not genotype dependent and suggests that increased stomatal density does not improve plant productivity in a lab environment.

Reduced stomatal density is associated with increased stomatal size, therefore it is theoretically possible for *fkf1-t* to optimize stomatal conductance with reduced stomatal density. Fewer, larger stomata with increased stomatal conductance could allow plants under well-watered growth conditions to increase CO_2_ uptake and its fixation into biomass. However, elevated stomatal conductance simultaneously increases photosynthetic and transpiration rates. Maximum water use efficiency requires a compromise between photosynthesis and transpiration so that CO_2_ uptake is maximized while water loss is minimized. Future studies measuring stomatal size and conductance of *fkf1-t* may reveal if the genotype has optimized the compromise between photosynthesis and transpiration. The timing and degree of stomatal aperture movement in response to environment may also impact gas exchange. Stomatal conductance is a function of stomatal density, stomatal aperture, and stomatal size [52]. Confirming a new role of FKF1 in organizing stomatal architecture to influence stomatal conductance would be significant because red-light mediated phytochromes and blue-light mediated cryptochromes are the only photoreceptors known to influence stomatal architecture [53].

## Limitations

Limitations of the methodology described in this paper include imaging only the above-ground portion of vegetative biomass. Root biomass was not accounted for. It was deemed appropriate to trim inflorescences of *Arabidopsis thaliana* prior to imaging in an effort to increase imaging efficiency while avoiding biomass overestimations of inflorescences positioned too close to the lens [18]. This methodology inevitably leads to reproductive biomass being lost, a factor which must be considered when interpreting results. Previous field studies on biomass estimates and plant species have indicated Canopeo may be appropriate for use on upright growing crops [33]. The performance of Canopeo for imaging upright growing crops in a controlled environment was not tested in this study.

## Conclusions

Results from this study demonstrate the method’s overall value for detecting vigor, partitioning, and architecture traits directly related to biomass accumulation over the lifecycle of *Arabidopsis thaliana* mutants in a controlled environment. Precision reduction of background noise in ImageJ prior to batch processing in Canopeo improved phenotyping of seedling biomass accumulation as compared to Canopeo’s built-in noise reduction algorithm. This suggests Canopeo’s built-in denoising algorithm may be improved by including compensation for image brightness. Investigating mutant genotypes for relationships between gas exchange, biomass accumulation, and senescence timing in response to environment offered insights into additional regulatory roles of the FKF1 gene.

Canopeo, which has already been shown to be effective for phenotyping biomass traits in the field [19,20], also provides a very simple method for estimating green biomass accumulation as grams across the plant lifecycle in a controlled environment. The present study demonstrates the use of Canopeo for model plants and suggests its value for low-cost and low-labor phenotyping of broadleaved specialty crops in both lab and greenhouse settings. Canopeo also served as a tool for phenotyping stomatal architecture, thereby guiding hypotheses for additional physiological roles of the FKF1 gene. By assisting in the formation of hypotheses, this methodology demonstrates its value for designing further experiments and budgeting high-tech research equipment. Future research may include determining Canopeo’s threshold of sensitivity for 1) phenotyping biomass related traits of broadleaved specialty crops in lab and greenhouse environments, 2) differentiating plant genotypes with similar phenotypic characteristics, and 3) imaging on a variety of backgrounds. The value of adapting Canopeo for lab use is that those with limited experience and resources have access to phenotyping technology that is quick, accurate, and cost-efficient in a controlled environment setting.

## Supporting information

S1 Graphical abstract (TIF)

S1 Data (XLSX)

S1 Fig y-intercept equation for calibration of Canopeo measurements. (PDF)

S1 Table LS-means tables. (PDF)

S2 Table Regression analysis of Fig 7. (PDF)

S3 Table Regression analysis of Fig 8 and Underlying data. (PDF)
