# Supplementary figures and images for: Canopeo app as image-based phenotyping tool in controlled environment utilizing Arabidopsis mutants
Source: PLoS One. 2024 Mar 21;19(3):e0300667. doi: 10.1371/journal.pone.0300667 (PMC10957076; doi:10.1371/journal.pone.0300667)

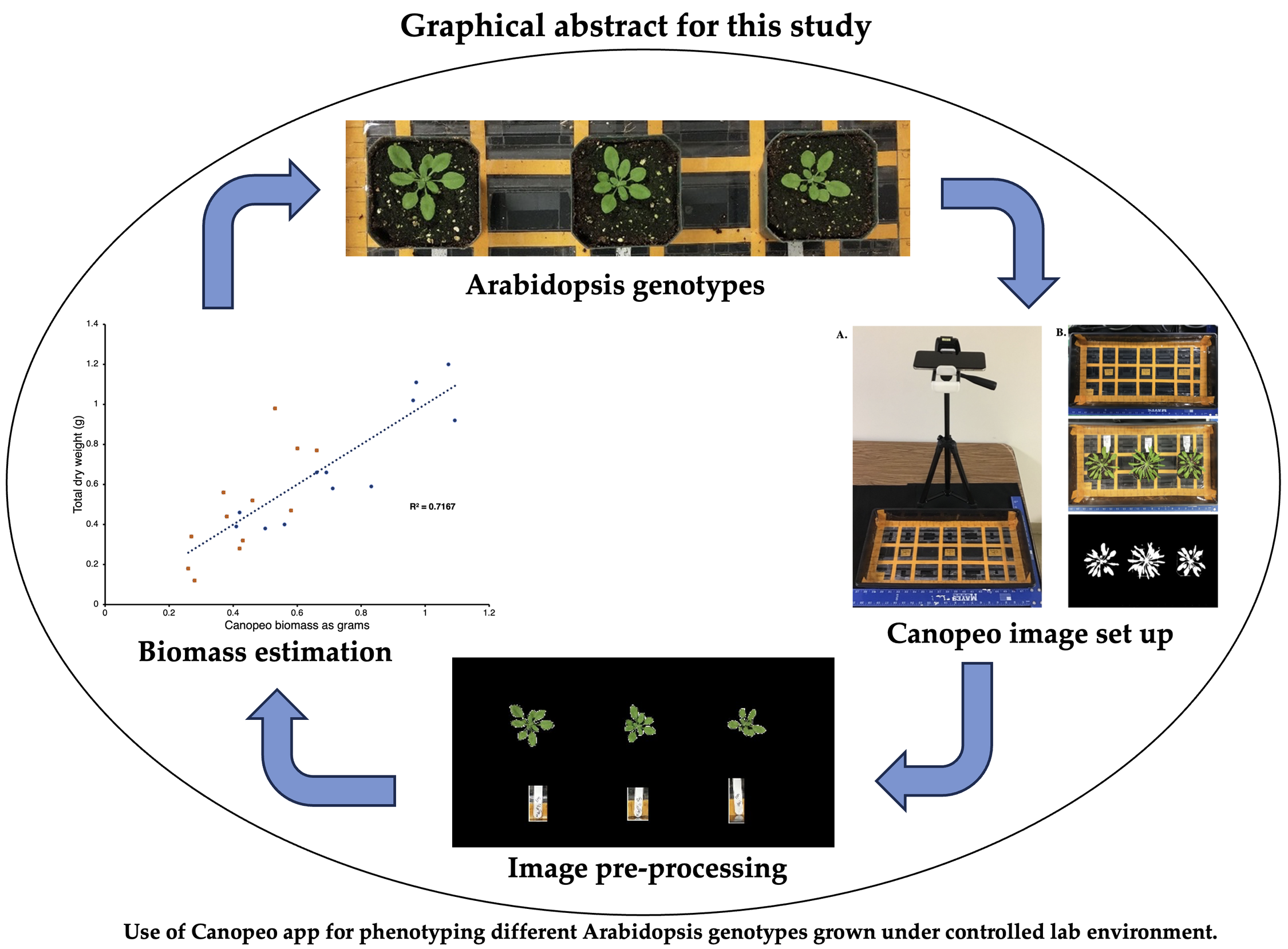

Supplement: S1 Graphical abstract — (TIF) [file pone.0300667.s001.tif]
