# Supplementary material for: Canopeo app as image-based phenotyping tool in controlled environment utilizing Arabidopsis mutants
Source: PLoS One. 2024 Mar 21;19(3):e0300667. doi: 10.1371/journal.pone.0300667 (PMC10957076; doi:10.1371/journal.pone.0300667)
Supplement: S1 Fig — (PDF) [file pone.0300667.s003.pdf]

Supplementary Figure S1

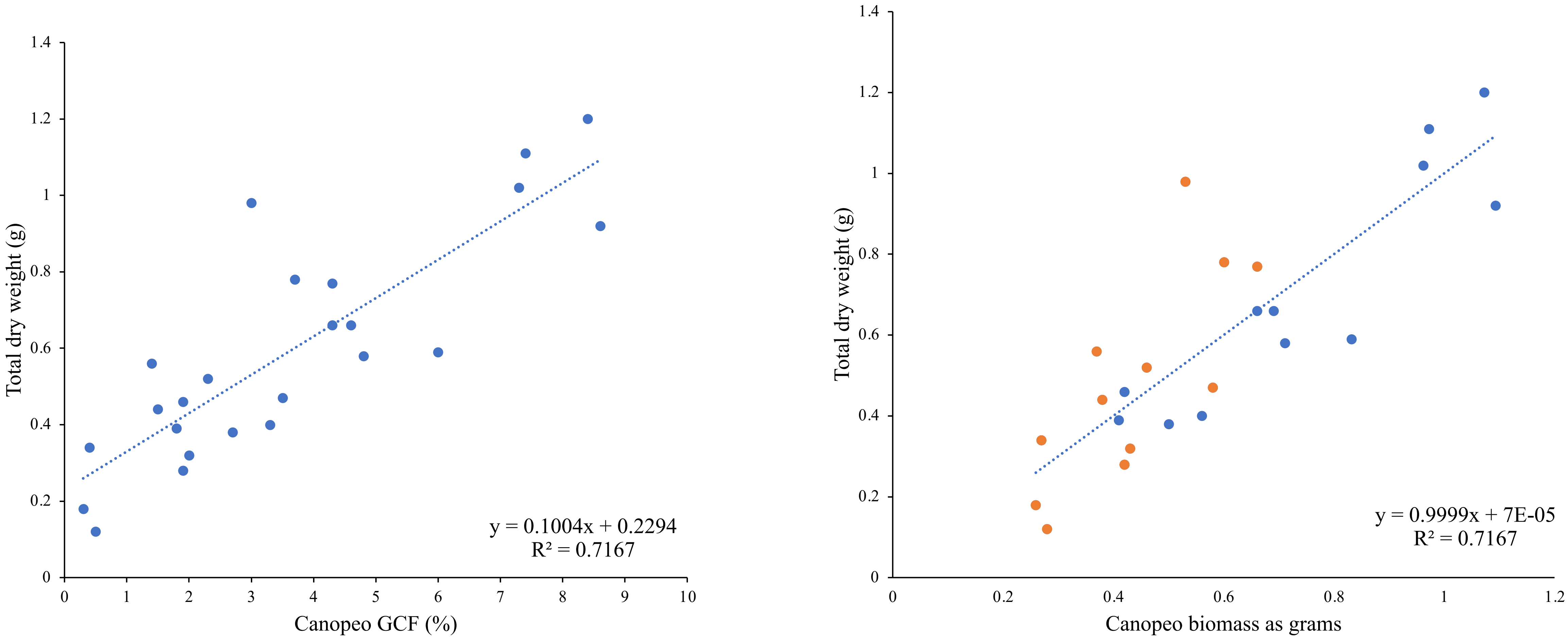

Supplementary Figure S1. Calibration of Canopeo GCF to estimate green biomass accumulation as grams.
Canopeo measurements of GCF were converted to estimate green biomass accumulation as grams by using the first y-intercept equation obtained. To convert measurements to estimate biomass, GCF values were input as the x-variable. The coefficient of determination (0.72) remains the same.
