# Supplementary material for: Canopeo app as image-based phenotyping tool in controlled environment utilizing Arabidopsis mutants
Source: PLoS One. 2024 Mar 21;19(3):e0300667. doi: 10.1371/journal.pone.0300667 (PMC10957076; doi:10.1371/journal.pone.0300667)
Supplement: S1 Table — (PDF) [file pone.0300667.s004.pdf]

Supplementary Table S1. List of LS-means tables

Table S1. LS-means tables for each experimental replicate demonstrated six out of twelve tables had significant ranking of *fkf1-t* based on numerical order and rank alone, excluding alphabetical groupings

LS-means tables	Line	Week 3	Week 4
		Estimate biomass accumulation (g)	
1-2	fkf1-t	0.26	0.35
	Col-0	0.25	0.34
	FKF1-OE	0.26	0.35
3-4	fkf1-t	0.24	0.26
	Col-0	0.24	0.25
	FKF1-OE	0.24	0.26
5-6	fkf1-t	0.28	0.48
	Col-0	0.26	0.40
	FKF1-OE	0.27	0.43
7-8	fkf1-t	0.25	0.36
	Col-0	0.25	0.35
	FKF1-OE	0.25	0.34
9-10	fkf1-t	0.32	0.57
	Col-0	0.30	0.53
	FKF1-OE	0.33	0.53
11-12	fkf1-t	0.25	0.46
	Col-0	0.25	0.44
	FKF1-OE	0.25	0.40

Table 1. Estimate biomass accumulation as grams by week in seven experiments comparing *fkf1-t*, Col-0, and FKF1-OE plants with twelve biological replications per experiment. *fkf1-t* demonstrated above average seedling vigor in four out of seven experiments, along with a later onset of senescence compared to the other genotypes. On average, Col-0 appeared to enter senescence later than FKF1-OE. Experiment 7 is an analysis of three replicate experiments. Least Square-means statistical analysis was performed using SAS GLIMMIX.
