# Supplementary material for: Canopeo app as image-based phenotyping tool in controlled environment utilizing Arabidopsis mutants
Source: PLoS One. 2024 Mar 21;19(3):e0300667. doi: 10.1371/journal.pone.0300667 (PMC10957076; doi:10.1371/journal.pone.0300667)
Supplement: S2 Table — (PDF) [file pone.0300667.s005.pdf]

Supplementary Table S2. Regression analysis of Figure 7

SUMMARY OUTPUT

Regression Statistics_fkf1-t 8 week	
Multiple R	0.767805775
R Square	0.589525709
Adjusted R Square	0.557950763
Standard Error	23.82399631
Observations	15

ANOVA

	df	*SS*	*MS*	*F*	*Significance F*
Regression	1	10597.15693	10597.1569	18.67068016	0.000830411
Residual	13	7378.576404	567.5828		
Total	14	17975.73333			

	Coefficients	*Standard Error*	*t Stat*	*P-value*	*Lower 95%*	*Upper 95%*	*Lower 95.0%*	*Upper 95.0%*
Intercept	250.9479558	21.89893351	11.4593688	3.62545E-08	203.6381862	298.257725	203.6381862	298.2577254
X Variable 1	-112.0734643	25.93717828	-4.3209582	0.000830411	-168.1073313	-56.039597	-168.1073313	-56.03959736

SUMMARY OUTPUT

Regression Statistics_fkf1-t 10 week low light	
Multiple R	0.702094245
R Square	0.492936329
Adjusted R Square	0.453931431
Standard Error	26.4790793
Observations	15

ANOVA

	df	*SS*	*MS*	*F*	*Significance F*
Regression	1	8860.892003	8860.892	12.63780595	0.003522729
Residual	13	9114.84133	701.141641		
Total	14	17975.73333			

	Coefficients	*Standard Error*	*t Stat*	*P-value*	*Lower 95%*	*Upper 95%*	*Lower 95.0%*	*Upper 95.0%*
Intercept	301.1062852	40.24023199	7.48271743	4.61242E-06	214.1725493	388.040021	214.1725493	388.0400211
X Variable 1	-125.7393901	35.3700365	-3.5549692	0.003522729	-202.1517083	-49.327072	-202.1517083	-49.32707183

SUMMARY OUTPUT

Regression Statistics_fkf1-t 10 week high light	
Multiple R	0.794022102
R Square	0.630471099
Adjusted R Square	0.607375543
Standard Error	28.01553862
Observations	18

ANOVA

	df	*SS*	*MS*	*F*	*Significance F*
Regression	1	21425.68465	21425.6846	27.29837249	8.34091E-05
Residual	16	12557.92647	784.870404		
Total	17	33983.61111			

	Coefficients	*Standard Error*	*t Stat*	*P-value*	*Lower 95%*	*Upper 95%*	*Lower 95.0%*	*Upper 95.0%*
Intercept	289.7772666	20.33702861	14.2487515	1.6469E-10	246.6646919	332.889841	246.6646919	332.8898413
X Variable 1	-120.5871993	23.07984196	-5.2247844	8.34091E-05	-169.5142785	-71.66012	-169.5142785	-71.66011998

SUMMARY OUTPUT

Regression Statistics_Col-0 8 week	
Multiple R	0.067154987
R Square	0.004509792
Adjusted R Square	-0.078447725
Standard Error	36.28973218
Observations	14

ANOVA

	df	*SS*	*MS*	*F*	*Significance F*
Regression	1	71.59263074	71.5926307	0.054362672	0.819568377
Residual	12	15803.33594	1316.94466		
Total	13	15874.92857			

	Coefficients	*Standard Error*	*t Stat*	*P-value*	*Lower 95%*	*Upper 95%*	*Lower 95.0%*	*Upper 95.0%*
Intercept	163.7668972	119.1794047	1.37412079	0.194525918	-95.90271884	423.436513	-95.90271884	423.4365133
X Variable 1	-53.81110478	230.792404	-0.233158	0.819568377	-556.6645557	449.042346	-556.6645557	449.0423461

SUMMARY OUTPUT

Regression Statistics_Col-0 10 week low light	
Multiple R	0.511769589
R Square	0.261908112
Adjusted R Square	0.200400455
Standard Error	31.24787459
Observations	14

ANOVA

	df	*SS*	*MS*	*F*	*Significance F*
Regression	1	4157.772578	4157.77258	4.25813832	0.061387039
Residual	12	11717.15599	976.429666		
Total	13	15874.92857			

	Coefficients	*Standard Error*	*t Stat*	*P-value*	*Lower 95%*	*Upper 95%*	*Lower 95.0%*	*Upper 95.0%*
Intercept	-14.44069853	73.41584945	-0.1966973	0.847356415	-174.4000932	145.518696	-174.4000932	145.5186961
X Variable 1	263.6716274	127.7772442	2.0635257	0.061387039	-14.73107156	542.074326	-14.73107156	542.0743264

SUMMARY OUTPUT

Regression Statistics_Col-0 10 week high light	
Multiple R	0.306717446
R Square	0.094075591
Adjusted R Square	0.037455316
Standard Error	168.2094695
Observations	18

ANOVA

	df	*SS*	*MS*	*F*	*Significance F*
Regression	1	47011.68977	47011.6898	1.661517727	0.215723909
Residual	16	452710.8102	28294.4256		
Total	17	499722.5			

	Coefficients	*Standard Error*	*t Stat*	*P-value*	*Lower 95%*	*Upper 95%*	*Lower 95.0%*	*Upper 95.0%*
Intercept	117.5211095	148.632751	0.79068112	0.440693264	-197.5662469	432.608466	-197.5662469	432.608466
X Variable 1	378.4263257	293.5816125	1.28899873	0.215723909	-243.9388903	1000.79154	-243.9388903	1000.791542
