# Supplementary material for: Canopeo app as image-based phenotyping tool in controlled environment utilizing Arabidopsis mutants
Source: PLoS One. 2024 Mar 21;19(3):e0300667. doi: 10.1371/journal.pone.0300667 (PMC10957076; doi:10.1371/journal.pone.0300667)
Supplement: S3 Table — (PDF) [file pone.0300667.s006.pdf]

Supplementary Table S3. Regression analysis of Figure 8

SUMMARY OUTPUT

Regression Statistics	
Multiple R	0.56058061
R Square	0.31425062
Adjusted R S	0.28159589
Standard Err	74.7140312
Observations	23

ANOVA

	df	*SS*	*MS*	*F*	*Significance F*
Regression	1	53719.7965	53719.7965	9.62343284	0.00539603
Residual	21	117225.916	5582.18646		
Total	22	170945.712			

	Coefficients	*Standard Error*	*t Stat*	*P-value*	*Lower 95%*	*Upper 95%*	*Lower 95.0%*	*Upper 95.0%*
Intercept	320.308137	23.1865078	13.8144191	5.2064E-12	272.089154	368.52712	272.089154	368.52712
X Variable 1	-1.07E-05	3.4487E-06	-3.1021658	0.00539603	-1.787E-05	-3.526E-06	-1.787E-05	-3.526E-06
